# Early differential diagnosis of ankylosing spondylitis among patients with low back pain in primary care

**DOI:** 10.1186/s12875-020-01161-6

**Published:** 2020-05-16

**Authors:** A. Riis, J. L. Olesen, J. L. Thomsen

**Affiliations:** 1 Center for General Practice at Aalborg University, Fyrkildevej 7, 1. Sal, 9220 Aalborg Ø, Denmark; 2 Department of Physiotherapy, University College of Northern Denmark (UCN), Selma Lagerløfs Vej 2, 9220 Aalborg Ø, Denmark

**Keywords:** Early diagnosis, Spondylitis Ankylosing, Low Back pain, Primary health care

## Abstract

Diagnosing and treating low back pain (LBP) is a worldwide major primary care challenge in which a differential diagnosis between non-specific LBP and conditions with a known pathology is essential for choosing the optimal treatment strategy. The time required for the diagnosis of a condition such as ankylosing spondylitis (AS) was previously found too long. However, a recently published paper by Bashir et al. found that distinct episodes of axial pain separated by more than 6 months seem more predictive than currently applied characteristics in reaching an early diagnosis of AS.

## Background

With a global one-month point prevalence of 23.2%, low back pain (LBP) is a major health challenge across cultures [1]. LBP is the leading cause of work disability and years lived with disability (YLDs) worldwide [2, 3].

Clinical diagnosis of LBP based on the patients’ history and clinical examinations is the key initial assessment by the first-line assessor of LBP—often the general practitioner (GP). This triage determines the subsequent diagnostic workup and informs the future treatment plan for the patient, including involvements of allied health care providers and medical specialist referrals [4]. The purpose of diagnostic triage of LBP is to allocate patients to one of three broad categories: specific spinal pathology (< 1% of cases), radicular syndrome (∼ 5–10% of cases), or non-specific LBP (90–95% of cases), where non-specific LBP is identified by the exclusion of the first two first categories [5]. Most cases are, therefore, considered unrelated to specific known spinal abnormalities [6]. Patients with LBP constitute a group with a large variation in the manifestations, possible bio-psycho-social causes, precipitating and maintaining factors, course, and prognosis [7]. Most patients with LBP appear to follow a particular pain trajectory over long periods and do not have frequently recurring or widely fluctuating patterns [8]. However, a subgroup constituting 13% of patients can be classified as having a fluctuating pain trajectory [9]. Patients belonging to the fluctuating pain trajectory show small improvements in functional capability [9]. Furthermore, their psychological status is without improvement after 12 months, with the proportion of patients classified as depressed remaining constant (27–30%). Almost half had experienced pain for more than 3 years, and a third was still consulting their general practitioner about back pain at the 12-month follow-up [9]. A newly published paper by Bashir et al. provides findings that might explain why patients with non-specific fluctuating pain have worse prognoses compared to patients with non-specific constant pain [10].

## Main text

Ankylosing Spondylitis (AS) is considered a relatively rare diagnosis in general practice [11]. However, in populations such as in the UK, with a high proportion of HLA-B27 positive in the population, the prevalence of AS among patients with LBP is up to 5% [11]. AS is a condition with a long time between an initial consultation for LBP before receiving an AS diagnosis [12]. The diagnostic delay in AS has previously been found unacceptably long, with females, younger patients, HLA-B27 negative, or patients with psoriasis having the longest diagnostic delay [13]. Early symptoms of AS besides LBP are stiffness and fatigue. These are considered non-specific symptoms and are similar to symptoms reported among patients with non-specific LBP [14]. Other diagnostic characteristics of AS, such as pain in the second half of the night and relief of pain and stiffness by exercise, are also often reported by patients with non-specific LBP [4, 15]. Nonsteroidal anti-inflammatory drugs can provide pain relief of AS, but no established diagnostic serum biomarkers allow the identification of AS in patients with early LBP [16].

Among the large group of patients with non-specific LBP, recent studies have found fear avoidance and other psychosocial factors predictive of worse outcomes when experiencing LBP [17, 18]. While including these factors in diagnostic screening tools provides some diagnostic information, it is important to stress the potential for misclassification of patient risk when using the available screening tools [19], thus making the clinical difference between non-specific LBP and AS hard to detect, which can increase delay for correct diagnosis.

Good early predictors for AS have previously been difficult to identify [20]. However, a newly published paper, including 74 patients with AS, concluded that distinct episodes of axial pain separated by more than 6 months are frequently observed before an AS diagnosis [10]. These episodes of pain among patients with LBP are highly associated with later receiving an AS diagnosis (OR 12.7, 95% CI 4.7 to 34.6) [10]. Among patients later diagnosed with AS, recurrent episodes of LBP were an even more frequent finding than either large joint symptoms or tendon symptoms [10]. In this new study, distinct episodes of axial pain separated by more than 6 months seem more predictive than currently applied characteristics in reaching an early diagnosis of AS [10]. However, these findings need to be duplicated in future research including larger study populations.

## Conclusion

A newly published paper found that two distinct periods of axial pain is predictive of receiving an AS diagnosis. This can support clinicians in reaching earlier diagnoses of AS among patients with non-specific pain, and this finding is important to inform future research into the differential diagnosis of patients with fluctuating non-specific LBP.

## Data Availability

Not applicable.

## Abbreviations

AS: Ankylosing spondylitis
LBP: Low back pain
